# Structure of SALO, a leishmaniasis vaccine candidate from the sand fly *Lutzomyia longipalpis*

**DOI:** 10.1371/journal.pntd.0005374

**Published:** 2017-03-09

**Authors:** Oluwatoyin A. Asojo, Alan Kelleher, Zhuyun Liu, Jeroen Pollet, Elissa M. Hudspeth, Wanderson C. Rezende, Mallory Jo Groen, Christopher A. Seid, Maha Abdeladhim, Shannon Townsend, Waldione de Castro, Antonio Mendes-Sousa, Daniella Castanheira Bartholomeu, Ricardo Toshio Fujiwara, Maria Elena Bottazzi, Peter J. Hotez, Bin Zhan, Fabiano Oliveira, Shaden Kamhawi, Jesus G. Valenzuela

**Affiliations:** 1 Sabin Vaccine Institute and Texas Children’s Hospital Center for Vaccine Development, National School of Tropical Medicine, Baylor College of Medicine, Houston, Texas, United States of America; 2 Vector Molecular Biology Section, LMVR, National Institute of Allergy and Infectious Diseases, National Institutes of Health, Rockville, Maryland, United States of America; 3 Departamento de Parasitologia, Instituto de Ciências Biológicas, Universidade Federal de Minas Gerais, Belo Horizonte, Minas Gerais, Brazil; 4 Department of Biology, Baylor University, Waco, Texas, United States of America; University of Notre Dame, UNITED STATES

## Abstract

**Background:**

Immunity to the sand fly salivary protein SALO (**S**alivary **A**nticomplement of *Lutzomyia* ***lo****ngipalpis*) protected hamsters against *Leishmania infantum* and *L*. *braziliensis* infection and, more recently, a vaccine combination of a genetically modified *Leishmania* with SALO conferred strong protection against *L*. *donovani* infection. Because of the importance of SALO as a potential component of a leishmaniasis vaccine, a plan to produce this recombinant protein for future scale manufacturing as well as knowledge of its structural characteristics are needed to move SALO forward for the clinical path.

**Methodology/Principal findings:**

Recombinant SALO was expressed as a soluble secreted protein using *Pichia pastoris*, rSALO(P), with yields of 1g/L and >99% purity as assessed by SEC-MALS and SDS-PAGE. Unlike its native counterpart, rSALO(P) does not inhibit the classical pathway of complement; however, antibodies to rSALO(P) inhibit the anti-complement activity of sand fly salivary gland homogenate. Immunization with rSALO(P) produces a delayed type hypersensitivity response in C57BL/6 mice, suggesting rSALO(P) lacked anti-complement activity but retained its immunogenicity. The structure of rSALO(P) was solved by S-SAD at Cu-K_alpha_ to 1.94 Å and refined to *R*_*factor*_ 17%. SALO is ~80% helical, has no appreciable structural similarities to any human protein, and has limited structural similarity in the C-terminus to members of insect odorant binding proteins. SALO has three predicted human CD4^+^ T cell epitopes on surface exposed helices.

**Conclusions/Significance:**

The results indicate that SALO as expressed and purified from *P*. *pastoris* is suitable for further scale-up, manufacturing, and testing. SALO has a novel structure, is not similar to any human proteins, is immunogenic in rodents, and does not have the anti-complement activity observed in the native salivary protein which are all important attributes to move this vaccine candidate forward to the clinical path.

## Author summary

Immunity to sand fly salivary proteins has been shown to confer protection against leishmaniasis in rodent models. Recombinant salivary protein SALO (**S**alivary **A**nticomplement of *Lutzomyia* ***lo****ngipalpis*) was shown to protect hamsters against the fatal outcome of visceral leishmaniasis caused by *Leishmania infantum* and to protect against cutaneous leishmaniasis caused by *Leishmania braziliensis*. Because of the potential use of this sand fly salivary protein as a component of a vaccine against human visceral leishmaniasis further characterization of SALO needs to be performed as well as a development plan for future scale manufacturing. In this work we present the successful expression and purification of recombinant SALO using *Pichia pastoris*. SALO from insect saliva inhibits the classical pathway of complement, an activity that may interfere with its role as a vaccine candidate. Here we show that recombinant SALO produced from *Pichia pastoris*, rSALO(P), does not have the anti-complement activity, and antibodies against rSALO(P) inhibit the anti-complement activity of sand fly saliva, suggesting that the overall structure of SALO is conserved regardless of expression source. The high-resolution structure of SALO was determined by single atom anomalous dispersion and refined to 1.94 Å. SALO is a small compact helical protein with no appreciable structural similarity to anti-complement or any reported mammalian protein structures. Therefore, SALO is a feasible candidate to be incorporated as a component of an effective anti-*Leishmania* vaccine.

## Introduction

Sand flies are vectors of *Leishmania* parasites, causal agents of the neglected tropical disease (NTD) leishmaniasis, which is ranked among the most important NTDs in terms of global disease burden [1] and is re-emerging at alarming rates due to the ongoing conflicts in the Middle East and North Africa [2]. To date, there is no effective or licensed vaccine against human leishmaniasis, despite studies demonstrating the cost-effectiveness of developing such biotechnologies for use in resource-poor settings [3, 4].

Sand flies deliver saliva into the skin of the host while probing for a blood meal. The saliva of blood feeding arthropods, including sand flies, has a number of potent bioactive molecules, such as anticoagulants, vasodilators, and inhibitors of platelet aggregation that assist in acquiring blood meals. In the case of sand flies, some of these bioactive components also modify the immunological environment at the host skin site of bite, favoring the establishment of *Leishmania* infection in the host [5, 6]. Other biological activities of sand fly saliva have been reported and the proteins responsible for these effects have been identified [7], including Lundep, an endonuclease that destroys neutrophil traps [8]; salivary yellow proteins that bind biogenic amines [9]; and recently SALO (Salivary Anti-complement from *Lutzomyia* *lo**ngipalpis*), an inhibitor of the classical pathway of complement [10].

Although sand fly saliva was shown to exacerbate *Leishmania* infection, immunity to sand fly saliva protects against *Leishmania* infection [6, 11]. The protection is correlated to the induction of a Th1 cellular immune response. Recently, a number of salivary proteins have emerged as vaccine candidates against cutaneous leishmaniasis, including PpSP15[12] and Linb11 [13] in rodent models, and PdSP15 in non-human primates[14]. For visceral leishmaniasis, the salivary proteins LJM17 and LJL143 were shown to induce a Th1 immune response in dogs [15]. Immunization with DNA plasmid coding for the salivary protein SALO (formerly known as LJM19) was shown to protect hamsters against the fatal outcome of visceral leishmaniasis [16] and this protection was correlated with the induction of a Th1 cellular immune response [16]. Recently, a combination vaccine comprised of recombinant salivary protein SALO and a genetically modified *Leishmania donovani* resulted in a strong protection against visceral leishmaniasis [17], further suggesting SALO as an important component for a visceral leishmaniasis vaccine.

In this study we present the production and crystal structure of SALO genetically engineered in yeast as part of efforts to develop it as a recombinant vaccine for leishmaniasis.

## Methods

### Ethics statement

All animal procedures were reviewed and approved by the National Institute of Allergy and Infectious Diseases (NIAID) Animal Care and Use Committee, under animal protocol LMVR4E, and handled in accordance to the Guide for the Care and Use of Laboratory Animals and with the NIH OACU ARAC. Further, the animal protocol is in full accordance with ‘The guide for the care and use of animals’ as described in the US Public Health Service policy on Humane Care and Use of Laboratory Animals 2015 (http://grants.nih.gov/grants/olaw/references/phspolicylabanimals.pdf).

### Cloning, expression and purification of recombinant SALO in *Pichia pastoris*

DNA coding for SALO without signal peptide was codon optimized based on *Pichia pastoris* usage preference and subcloned into *Pichia* secretory expression vector pPICZαA (Invitrogen) using EcoRI/XbaI restriction sites. The correct insert sequence and reading frame of recombinant plasmid was confirmed by double-stranded sequencing using vector flanking primers α-factor and 3’AOX-1 and then transformed into *Pichia pastoris* X-33 by electroporation. The expression of rSALO(P) was induced with 0.5% methanol at 30°C for 72 hours and the highest expression clone was chosen for making seed stock with 20% glycerol. The large-scale expression of hexa histidine tagged rSALO(P) was induced with methanol in a 10L fermentor and purified by immobilized metal affinity chromatography (see S1 Text).

### Glycosidase treatment of recombinant proteins

Recombinant proteins rSALO(P) and rSALO(H) were treated with glycosidases using the Enzymatic DeGlycoMx Kit from QA-Bio (Palm Desert, CA), which contains a mixture of N-glycosidase F, sialidase, ß-galactosidase, glucosaminidase, and O-glycosidase following the manufacturer’s instructions. Control reactions were performed without glycosidases.

### Cloning and production of recombinant SALO in HEK293-F

Cloning, expression and purification of SALO was performed as previously described [10]. Briefly, DNA coding for SALO without the signal peptide and containing a C-terminal hexahistidine tag was synthesized by Eurofins genomics (Huntsville, AL). The synthesized gene was subcloned into the VR2001-TOPO expression vector. The transfection into HEK 293-F cells and expression of rSALO(H) was performed at the Protein Expression Laboratory at the Frederick National Laboratory for Cancer Research (Frederick, Maryland). The supernatant was recovered after 72 hours, concentrated, and buffer exchanged into PBS pH 7.4 using a 10K Amicon concentrator device (Millipore). The protein was purified by immobilized metal ion affinity chromatography in the same buffer and eluted with imidazole.

### Hemolytic assays

Hemolytic assays were performed as previously described [10]. Briefly, 1% normal human serum (NHS) in gelatin HEPES buffer with Ca^+2^ and Mg^+2^ (0.1% gelatin, 5mM HEPES, 145 mM NaCl, 0.15 mM CaCl_2_, 0.5 mM MgCl_2_, pH 7.4) containing SALO was added to antibody sensitized sheep erythrocytes (1 x 10^7^ cells) and incubated 30 min at 37°C. After adding 250 μl ice cold saline (0.9% NaCl) the sample was centrifuged and the absorbance at 414nm was measured.

### Blockage of SGH anti-complement activity by rSALO(P) antiserum

Hemolytic assays using SGH or rSALO(P) in the presence of the rSALO(P) antiserum were performed as described above, but prior to mixing the SGH (0.5 salivary gland pairs) or rSALO(P) (final concentration 0.1 μM) with the NHS in GVB^2+^ (5 mM Veronal, 145 mM NaCl, 0.15 mM CaCl_2_, 0.5 mM MgCl_2_, 0.025% NaN_3_ and 0.1% gelatin, pH 7.3), the inhibitors were incubated with 12.5 μl of different dilutions of the antiserum (1:10; 1:100; 1:1000 or 1:10000, in PBS). A control experiment using antiserum diluted 1:10 with red blood cells, was performed to determine any possible hemolytic effects of the cells hemolysis.

### Sand fly Salivary Gland Homogenate (SGH) preparations

Salivary glands were dissected from *Lu*. *longipalpis* sand flies obtained from the Vector Molecular Biology Section, LMVR, NIAID, NIH as previously described [18].

### Antibodies to rSALO

Six to eight weeks old female Balb/c mice were injected intradermally in the ear three times every 15 days with 2 μg of rSALO(P) mixed (1:1 volume) with Magic Mouse Adjuvant (Creative Diagnostics, Shirley, NY) as recommended by the manufacturer. Fifteen days after the last inoculation, blood was collected to obtain the rSALO antiserum.

### Immunization of mice with rSALO and DTH measurements

Six to eight weeks old female Balb/c mice were injected intradermally in the right ear three times every 15 days with 2 μg of rSALO(P) (without adjuvant). The endotoxin level of rSALO(P) was 0.00127 Endotoxin Units per injection. Delayed type hypersensitivity response or skin immune response was measured in the ear of C57Bl/6 mice as previously described [19, 20]. The mouse ear thickness and redness were used as an indicator of a cell-mediated immune response to rSALO(P)[19, 20]. Briefly, ear thickness from the from the dorsal to the ventral portion of the ear was measured using a Digital Vernier caliper (Mitutoyo Corp.) at 24 and 48 h following intradermal injection of rSALO(P). Measurements were taken for five mice in each group and repeated at least twice.

### Statistical analysis

Statistical analysis was performed using the GraphPad Prism software. Multiple groups were analyzed using one-way analysis of variance followed by Tukey's multiple-comparison test.

### Size-Exclusion Chromatography and Multi-Angle Light Scattering (SEC-MALS)

The average molecular weight of the SALO protein was determined by SEC-MALS. The system consisted of an Agilent 1260 Infinity series HPLC, coupled with a UV detector (Agilent), a miniDAWN triple-angle light-scattering detector (Wyatt Technology), and an Optilab rEX differential Refractive Index (dRI) detector (Wyatt Technology). 40 μg of SALO was loaded into a TSK gel Super SW2000 column (TOSOH Biosciences, King of Prussia, PA) and eluted at 0.35 ml/min isocratically with Tris-HCL pH 8 for 30 min. Protein constants were 0.185 mL/g and 0.911 mL/(mg∙cm) for dRI and UV detectors, respectively. Data collection and analysis was done with Wyatt’s ASTRA 6.1.1 software.

### Crystallization and data collection

rSALO(P) was buffer exchanged and concentrated to 24 mg/ml in 50 mM Tris HCl pH 8.0 using a 5K MW cutoff centrifugal concentrating device (Millipore). The initial protein concentration was confirmed by measurement of OD_280_ prior to setting up crystallization experiments. Crystallization conditions were screened using commercial screens from Hampton Research at 298K. Crystals were grown by vapor diffusion in sitting drops, which were equilibrated against well containing 0.5 ml crystallization solution. Drops were prepared by mixing 1.5 μl of protein solution with an equal volume of crystallization solution. No crystals were obtained for protein produced in mammalian cells, possibly because of the presence of the N-terminus vector derived sequence. rSALO(P) crystallized within 16 hours from a precipitant solution containing 0.02M calcium chloride, 30% v/v MPD and 0.1 M sodium acetate pH 4.6. Larger crystals with dimensions 0.8 mm X 0.5 mm X 0.3 mm were obtained within 48 hours by setting up larger drops using a ratio of 4 μl of protein to 1.5 μl of the same precipitant solution.

Since crystals grew in solutions that contained adequate cryoprotectant, they were flash-cooled directly in a stream of N_2_ gas at 113 K prior to collecting diffraction data. X-ray diffraction data were collected at the Baylor College of Medicine core facility (Rigaku HTC detector, Rigaku FR-E+ SuperBright microfocus rotating anode generator, with VariMax HF optics) using the Crystal Clear (d*trek) package [21]. Data was integrated using MosFLM and scaled with SCALA [22]. Crystallographic data is shown in Table 1.

**Table 1:** Statistics for data collection and model refinement.

Data Collection	PDB entry 4lu2
X-ray Source	Rigaku FR-E+
Detector	Rigaku HTC
Wavelength	0.15418 nm
Space group	*P*4_2_
Cell dimensions	a = b = 65.28Å, c = 59.06Å α = β = γ = 90.00°
Resolution (Å)	46.7–1.94 (2.0–1.94)
Number of total reflections	254520 (35701)
Number of unique reflections	12748 (1821)
^*†*^*R*_*merge*_	8.8 (52.1)
*I/σ(I)*	23.4 (6.0)
Completeness (%)	100 (100)
^†^Redundancy	20.0 (19.6)
Mn(I) half-set correlation CC(1/2)	0.998 (0.956)
Average Mosaicity	0.7
**Refinement (PHENIX)**	
Resolution (Å)	23.44–1.94 (2.23–2.10)
Percentage Data completeness	99.5 (99.4)
*R*_*Free*_ test set	684 reflections (5.51%)
Wilson B-factor (Å^2^)	29.5
Anisotropy	
No. of non-H protein atoms	1572
No. of water molecules	18
^a^*R*_*work*_	0.167 (0.134)
^b^*R*_*Free*_	0.188 (0.153)
Correlation coefficient *F*_*o*_*-F*_*c*_	0.94 (0.96)
Average B-factors (Å^2^)	31.3
Protein (Å^2^)	29.2
Water and other small molecules (Å^2^)	40.5
r.m.s. deviations	
Bond lengths (Å)	0.009
Bond angles (°)	1.074
MolProbity analysis	
Ramachandran outliers	0%
Ramachandran favored	97.3%
Rotamer outliers	1.7%
C-beta deviations	0
Clashscore	0.96

### Structure determination

The structure of rSALO(P) was solved using single-wavelength anomalous dispersion with the anomalous signal from sulfur at Cu-K_alpha_ wavelength. FA values were calculated using the program SHELXC [23]. Based on an initial analysis of the data, the maximum resolution for substructure determination and initial phase calculation was set to 1.94 Å. The location of 89 atoms (C, S, N, O) were automatically determined using the program SHELXD [23] and based on the results of this automated search 82.08% of the model was built using the program ARP/wARP [24, 25]. Since the difference between R_factor_ and R_free_ remained unreasonably high, the structure was subsequently refined in a lower symmetry space group with a dimer in the asymmetric unit. The final model was obtained by iterative manual model building cycles using the program Coot [26] followed by structure refinement with REFMAC5 [27][28] and PHENIX[29]. Structural figures were generated using PyMOL [30]. The refined coordinates and structure factors have been deposited in the RCSB protein databank under accession code 4LU2.

### Prediction of T cell epitopes

T-cell epitope was predicted for full SALO sequence using the program NetMHC II release 2.2 [31]. The program was set to default parameters that allow identification of 15-mer amino acid peptides with predicted binding affinity below 50 nM to MHC II alleles [32]. For predicted epitopes for the same MHC II allele with sequence length overlap higher than 50%, the peptide with the highest affinity score was kept. Graphs were built using in-house Perl scripts.

## Results

### SALO produced from *Pichia pastoris*, rSALO(P), is pure and monodisperse

**Fig 1:**
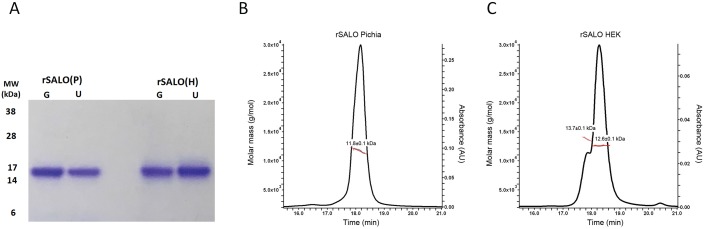
rSALO(P) is pure and monomeric in solution. A) Coomassie G-250 stained NuPAGE Bis-Tris gels under reducing conditions of 2 μg purified rSALO(P) or rSALO(H). Recombinant proteins were treated with glycosidades (G) or untreated (U) before SDS-PAGE analysis. B) SEC-MALS reveals that rSALO(P) is a monomer in solution with an estimated molecular weight of 11.8 kDa. C) SEC-MALS reveals that rSALO(H) is made up of different species.

SALO was produced in *Pichia pastoris* to establish a feasible process for a product and clinical development path. This includes testing the immunogenicity of this salivary protein and resolving its crystal structure. Recombinant SALO was expressed as a soluble protein with a vector derived EF on the amino terminus using *Pichia pastoris* after 72 hours of methanol induction. Typical yields of rSALO(P) by a single immobilized metal affinity chromatography purification step were ~ 1g/L, which is 500 times higher than the 2.0 mg per L for rSALO(H) produced in HEK293 cells. Purified rSALO(P) appeared to be ~99% pure (Fig 1A). The electrophoretic mobility of ~15kDa is likely due to the charge of the molecule and not due to post-translational modifications because the molecular weight of SALO determined by SEC-MALS (11.8 kDa) is close to the theoretical molecular mass of 11.9 kDa (Fig 1B). rSALO(P) elutes at 18.2 min as a single, monodisperse peak with a calculated molecular weight of 11.8 kDa (Fig 1B) which agrees with the theoretical molecular mass (11.9 kDa) of monomeric rSALO. Recombinant SALO produced from HEK cells, rSALO(H), elutes off the sizing column as two overlapping peaks (Fig 1C). The main peak at 18.3min is ~83.9% of all the protein components has a molecular weight of 12.6 kDa, and a minor overlapping peak (~15.5%) at 17.8 min with molecular weight of 13.7 kDa (Fig 1C). The theoretical molecular weight of rSALO(H) is 12.2 kDa.

Thus, rSALO(P) is pure and exclusively monomeric in solution (monodisperse), which will simplify the downstream process for the production of a recombinant biologic for clinical development.

### Antibodies against rSALO(P) inhibit the anti-complement activity of sand fly salivary gland homogenate

**Fig 2:**
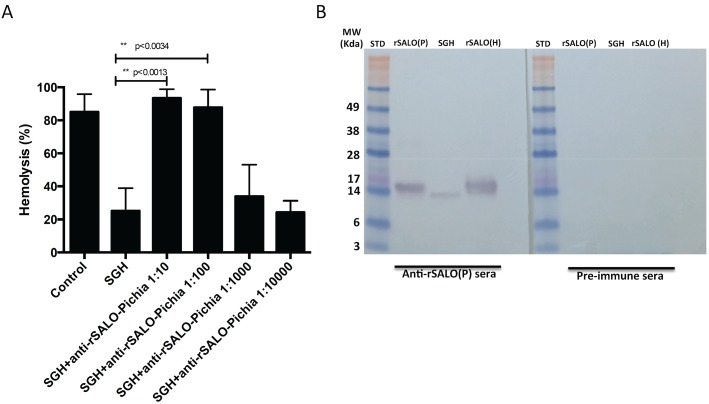
Antibodies against rSALO(P) block anti-complement activity present in the salivary glands of the sand fly *Lutzomyia longipalpis* (SGH). (A) Hemolytic assays using SGH (0.5 salivary gland pairs) in the presence of different dilutions of anti-rSALO(P) antibodies (1:10; 1:100; 1:1000 or 1:10000, in PBS). The data represents the mean ± standard deviation of three independent repetitions (ANOVA and Tukey test). Hemolysis was measured at 414nm. (B) Western blot showing rSALO(P) antibodies recognizing rSALO(P) (rSALO Pichia), native SALO from the salivary gland homogenate of *Lutzomyia longipalpis* (SGH), and rSALO(H) (rSALO HEK). SDS-PAGE was run under reducing conditions. Pre-immune sera was used as a control.

It was previously shown that rSALO(H) and SGH of *Lu*. *longipalpis* containing SALO inhibited the classical pathway of complement [10]. rSALO(P) did not inhibit the classical pathway of complement, in contrast to rSALO(H) which inhibits the classical pathway of complement (S1 Fig). Nevertheless, antibodies produced against rSALO(P) neutralized *Lu*. *longipalpis* SGH anti-complement activity in a dose dependent manner (Fig 2A). Importantly, antibodies raised against rSALO(P) recognized both rSALO(P) and rSALO(H), and a single band from *Lu*. *longipalpis* SGH, by Western blot (Fig 2B). Furthermore, rSALO(P) had similar immune recall responses as rSALO(H) (S2A and S2B Fig). C57BL/6 mice immunized with rSALO(P) or rSALO(H) induced an immune recall response as a form of a delayed-type hypersensitivity response (DTH) in the skin of mouse ear at 48 hours after the second and third immunization (S2A and S2B Fig). This immune response was not observed in control naïve animals or at the contralateral ear where rSALO(P) or rSALO(H) was not injected (S2A and S2B Fig).

### SALO is a small helical protein

**Fig 3:**
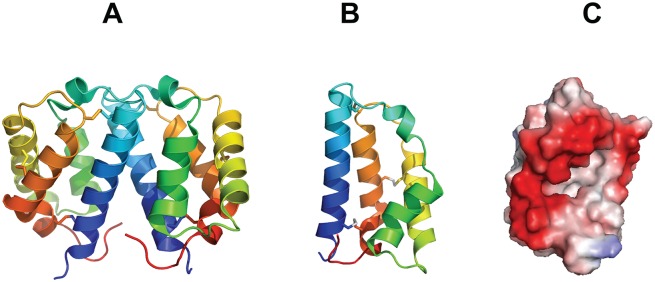
Crystal structure of SALO. A) Ribbon diagram of a SALO dimer with each monomer represented in rainbow colors from the N-terminus (blue) to the C-terminus (red). B) Ribbon diagram of a SALO monomer. C) Charge distribution on the surface of a SALO monomer in the same view as B.

The crystal structure of SALO was refined with a dimer in the asymmetric unit in the space group *P* 4_2_ with statistics shown in Table 1. We chose the dimer because refining the structure of SALO as a monomer in a higher symmetry space group (*P* 4_2_2_1_2), resulted in >12% difference between *R*_*Factor*_ and *R*_*Free*_ and increased disorder in loop regions. The SALO dimer (Fig 3A) appears to be crystallographic and PISA analysis shows no appreciable buried surface area at the dimer interface. SALO is ~80% alpha helix and ~20% loop. Each SALO monomer has an overall topology comprised exclusively of helices, stabilized by disulfide bonds and connected by short loops (Fig 3B). SALO also has large segregated exposed charged regions (Fig 3C).

### Predicted SALO T cell epitopes are exposed on the surface of the protein

**Fig 4:**
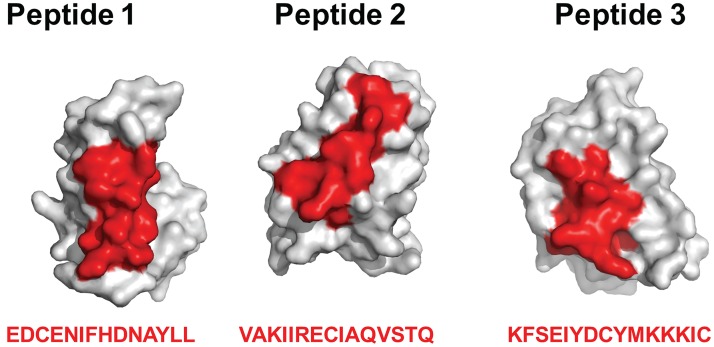
The three predicted T cell epitopes on SALO are located on exposed helices. The position of peptide sequences given at the bottom is shown in red on the SALO structural surface (gray).

There are three predicted human T cell epitopes in SALO, EDCENIFHDNAYLL (peptide 1), VAKIIRECIAQVSTQ (peptide 2) and KFSEIYDCYMKKKIC (peptide 3), (Table 2). All three epitopes are located on surface exposed helices (Fig 4).

**Table 2:** Predicted MHC class II T cell epitopes for the SALO protein in humans.

Allele	Initial Coordinate	Final Coordinate	Sequence	IC50 Affinity
DRB30101	1	15	SEDCENIFHDNAYLL	5.2
DRB11302	1	15	SEDCENIFHDNAYLL	16.5
DRB10301	2	16	EDCENIFHDNAYLLK	29.7
DRB10101	54	68	VAKIIRECIAQVSTQ	32.6
DRB11101	72	86	KFSEIYDCYMKKKIC	34.1
DPA10301-DPB10402	3	17	DCENIFHDNAYLLKL	34.5
DRB11501	3	17	DCENIFHDNAYLLKL	39.5
DRB10101	2	16	EDCENIFHDNAYLLK	41.3
DRB10802	54	68	VAKIIRECIAQVSTQ	46.2
DRB40101	52	66	EKVAKIIRECIAQVS	46.7
DRB50101	72	86	KFSEIYDCYMKKKIC	46.9

**Fig 5:**
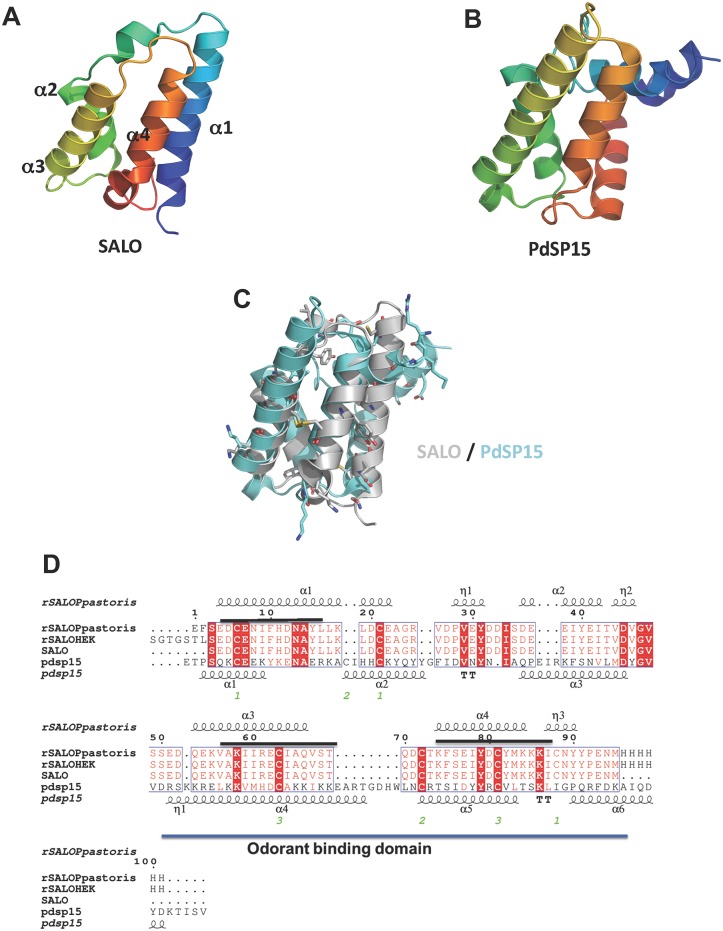
Comparison of SALO to PdSP15. (A) Ribbon diagram of a SALO monomer. (B) Ribbon diagram of pdsp15 (C) C-terminus of SALO (grey) superposes well with PdSP15 (aqua marine). (D) The amino acid sequence alignment comparing SALO to PdSP15 generated with *ESPript*3.0 [36] reveals a conserved C-terminal odorant-binding domain. The location of the three predicted T-cell epitopes are shown as black lines. Secondary-structure elements are as follows: α-helices (α), 3_10_-helices (η), β-strands (β) and β-turns (TT). Identical residues are shown on a red background; conserved residues are shown in red; and conserved regions are shown in blue boxes.

The structure of SALO is unique and could not have been predicted from any known protein structures. SALO is comprised entirely of helices and belongs to the all-alpha protein class with EF hand like fold. Pfam analysis using PDBSum (http://www.ebi.ac.uk/pdbsum/) reveals that the C-terminal of SALO (residues 50–105) contains the Pfam domain family PF01395, otherwise known as the odorant-binding domain of insect proteins. Members of this family have limited sequence identity and their proposed shared function is to bind insect pheromones or odorants [33, 34]. Additional studies are required to clarify if SALO can indeed bind odorants. The structure of the salivary protein PdSP15 from the sand fly *Phlebotomus duboscqi* has been reported, and like SALO, its structure is all helices connected by loops [35]. While SALO only shares 19.6% sequence similarity to PdSP15, their C-terminal odorant binding domains superpose quite well (Fig 5A–5C). Additionally, secondary structure alignment reveals a series of conserved residues including disulfide bonds that connect the central helices (Fig 5C & 5D). Interestingly, two of the three predicted T-cell epitopes (peptide 2 and peptide 3) are located in the structurally conserved C-terminus odorant-binding domain (Fig 5D). It remains unknown what roles these conserved residues play in odorant binding or the functions of SALO and similar proteins.

## Discussion

Immunization with sand fly salivary protein SALO protects against leishmaniasis (visceral and cutaneous), either as a DNA vaccine or as a recombinant protein [16, 37]. Furthermore, this vaccine candidate was recently shown to inhibit the classical pathway of complement [10]. To move this vaccine candidate towards the clinical path, we solved the structure of SALO and expressed it in *P*. *pastoris*. Further, we developed a process for pilot production of SALO. Results from the current work demonstrate the feasibility of expressing SALO in *P*. *pastoris* for future scale manufacturing. We previously showed that rSALO(H) has anti-complement activity. In this work, we reproduce this finding (S1 Fig) and demonstrate that rSALO(P) lacks anti-complement activity, and is monomeric. Interestingly, both native SALO from salivary glands and rSALO(H) form multi-species as previously shown by Western blot [10]. It is possible that some or one of these multiple species are required for anti-complement activity, and that the single species observed in rSALO(P) may not be the active form of the protein. Regardless, rSALO(P) has the desirable features of a vaccine candidate: it is monomeric, monodisperse and does not have anti-complement activity while retaining its immunogenicity. After immunization, both rSALO(H) and rSALO(P) induced a robust delayed hypersensitivity response in mice. Of note, though rSALO(P) lacks anti-complement activity, antibodies against it inhibit the anti-complement activity from *Lu*. *longipalpis* sand fly salivary gland homogenate, suggesting that the overall structure of SALO is conserved regardless of the expression source.

Our structural analyses also reveal that SALO does not share any appreciable tertiary or quaternary structural similarity to any known mammalian protein families. Furthermore, SALO is not found in any other insect vectors or other organisms [38], displaying appreciable sequence homology only to proteins found in New World sand flies of the genus *Lutzomyia and Nyssomyia* [39]. Interestingly, our current studies reveal that SALO and PdSP15, another salivary vaccine candidate that was previously shown to protect non-human primates against vector-transmitted *L*. *major* infection [14], have conserved structural features in their odorant binding protein domain, which contains two of the three predicted CD4^+^ T cell epitopes, strongly suggesting that the odorant binding protein domain may be relevant for their immunogenic properties. Both SALO and PdSP15 produce a robust cellular immune response that is protective against leishmaiasis [14, 16]. In light of our current findings, further studies are necessary to determine the importance of the odorant binding protein domain in the antigenicity of these salivary proteins.

In summary, this work demonstrates that rSALO(P) is suitable for further scale-up, manufacturing, and testing as a vaccine candidate against leishmaniasis. The structure of SALO is novel and unique to sand flies with no resemblance to any protein sequence or structure from humans. rSALO(P) retains its immunogenicity and importantly it lacks anti-complement activity, overcoming a potential obstacle for its development as a vaccine. The attributes of recombinant rSALO(P) and its feasibility for future large-scale production make this molecule an attractive target as a component of a *Leishmania* vaccine for humans.

## Supporting information

S1 Text Production of recombinant SALO. (DOCX)

S1 Fig Recombinant SALO produced in *Pichia pastoris* (rSALO[P]) does not inhibit the classical pathway of complement. rSALO (0.1μM) produced in Pichia (SALO Pichia) or rSALO (0.1μM) produced in HEK cells (SALO HEK) were tested on the classical pathway of complement using a hemolytic assay. Erythrocyte lysis was measured at 414nm. The data represents the mean plus the standard deviation of three independent experiments.
(PDF)

S2 Fig Skin immune response in rSALO immunized mice. C57bl/6 mice were immunized in the ear with 2 μg of recombinant SALO produced in *P*. *pastoris* (p) or SALO produced in HEK cells (m). Two weeks (A) or 4 weeks (B) after the last immunization 2 μg of recombinant protein was injected and induration and redness in the ear was measure at 48 hours. Naïve mice (control) were injected with PBS. The data represents the mean ± standard deviation of a representative experiment with 5 mice per group of two independent experiments (ANOVA and Tukey test).
(PDF)
