# Supplementary material for: Structure of SALO, a leishmaniasis vaccine candidate from the sand fly Lutzomyia longipalpis
Source: PLoS Negl Trop Dis. 2017 Mar 9;11(3):e0005374. doi: 10.1371/journal.pntd.0005374 (PMC5344329; doi:10.1371/journal.pntd.0005374)
Supplement: S1 Text — (DOCX) [file pntd.0005374.s001.docx]

**S1 Text. Production of recombinant SALO**

DNA coding for SALO minus the signal peptide with a C-terminal hexahistidine tag was codon optimized based on the yeast codon preference, synthesized by GenScript (Piscataway, NJ) and subsequently subcloned via *Eco*RI and *Xba*I restriction sites into pPICZ**α**A, a *Pichia pastoris* expression vector containing the yeast **α**-factor signal for secretory expression (Invitrogen). The open reading frame (ORF) and sequence was verified, by double-stranded DNA sequencing, using the vector flanking primers α-factor and 3’AOX1. The recombinant plasmid DNA was linearized with SacI digestion and then transformed into *P. pastoris* X33 strain by electroporation. The transformants were selected on zeocin-resistant YPD plates. Twenty transformed colonies were picked for induction of recombinant SALO protein with 0.5% methanol at 30°C for 72 hours and subsequently the colony with highest expression was used to make a research seed stock that was aliquoted and stored as 1mL SALO cell banks.

Fermentation was initiated by inoculation of a 10 L fermentation vessel (New Brunswick) as follows: 1 mL SALO cell bank was added to 1 L BMG media (100mM potassium phosphate pH 6.0, 1.34% YNB, 4x10^-5^% Biotin, 1% glycerol) and the flask incubated at 29 +/-1 °C with agitation at 250 +/-10 rpm overnight. After reaching the target OD_600_ (1.0–5.0), 250–500 mL of the seed culture was inoculated into 5 L of sterile basal salt media, BSM (85% 26.7 mL/L phosphoric acid, 0.93 g/L calcium sulfate, 18.2g/L potassium sulfate, 14.9 g/L magnesium sulfate, 4.13 g/L potassium hydroxide, and 40 g/L glycerol) containing 4.35 mL/L PTM1 trace salts (6.0 g/L copper sulfate, 0.08 g/L sodium iodide, 3.0 g/L manganese sulfate, 0.2 g/L sodium molybdate, 0.02 g/L boric acid), 0.5 g/L copper chloride, 20.0 g/L zinc chloride, (ZnCl_2_), 65.0 g/L iron (II) sulfate, 5 mL/L sulfuric acid) and 4.35 mL/L 0.02% d-biotin. The pH of BSM was adjusted to 5.00 +/− 0.2 prior to inoculation and maintained at this set point by the addition of 14% ammonium hydroxide. Fermentation set points used were 30 +/− 1 °C, dissolved oxygen 30%, air flow 0.5–1.0 SPLM, and agitation 500 rpm. Antifoam 204 was added to minimize foaming. Approximately 16 +/− 4 h after inoculation of the fermenter, a DO spike was observed due to the depletion of glycerol within the media. After observation of the DO spike, a 50% glycerol feed was initiated at a rate of 15 ml/hr/L for 6 hours. During the last two hours of the glycerol fed-batch stage, the pH was ramped from 5.0 to 6.0 and the temperature was ramped down from 30°C to 26°C. At the end of the fed-batch stage, agitation was increased to 700 rpm and a 100% methanol feed (containing 12 mL/L PTM1 Trace Salts and 12 ml/L 0.02% d-Biotin) was ramped from 1–11 ml/hr/L over an 8-h period. After the ramp period, the methanol feed was maintained at 11 ml/hr/L until the end of fermentation (approximately 72 h after methanol induction).

The induced fermentation culture was buffer exchanged to 1X PBS, pH 7.4 and concentrated with hollow fiber cartridge (3,000 MW cutoff, GE Health). Recombinant SALO (rSALO) was purified from the concentrated culture by immobilized affinity chromatography (IMAC) using HisTrap FF column (GE, Healthcare). The typical yield of purified rSALO from *P. pastoris* was 1g per L of culture. Coomassie G-250 (Simply Blue) stained NuPAGE Bis-Tris gels (Invitrogen) were used to assess the purity of the recombinant proteins. The purified rSALO was dialyzed to remove imidazole and stored at −80°C until used.
