# Supplementary material for: Structure of SALO, a leishmaniasis vaccine candidate from the sand fly Lutzomyia longipalpis
Source: PLoS Negl Trop Dis. 2017 Mar 9;11(3):e0005374. doi: 10.1371/journal.pntd.0005374 (PMC5344329; doi:10.1371/journal.pntd.0005374)
Supplement: S1 Fig — rSALO (0.1μM) produced in Pichia (SALO Pichia) or rSALO (0.1μM) produced in HEK cells (SALO HEK) were tested on the classical pathway of complement using a hemolytic assay. Erythrocyte lysis was measured at 414nm. The data represents the mean plus the standard deviation of three independent experiments. (PDF) [file pntd.0005374.s002.pdf]

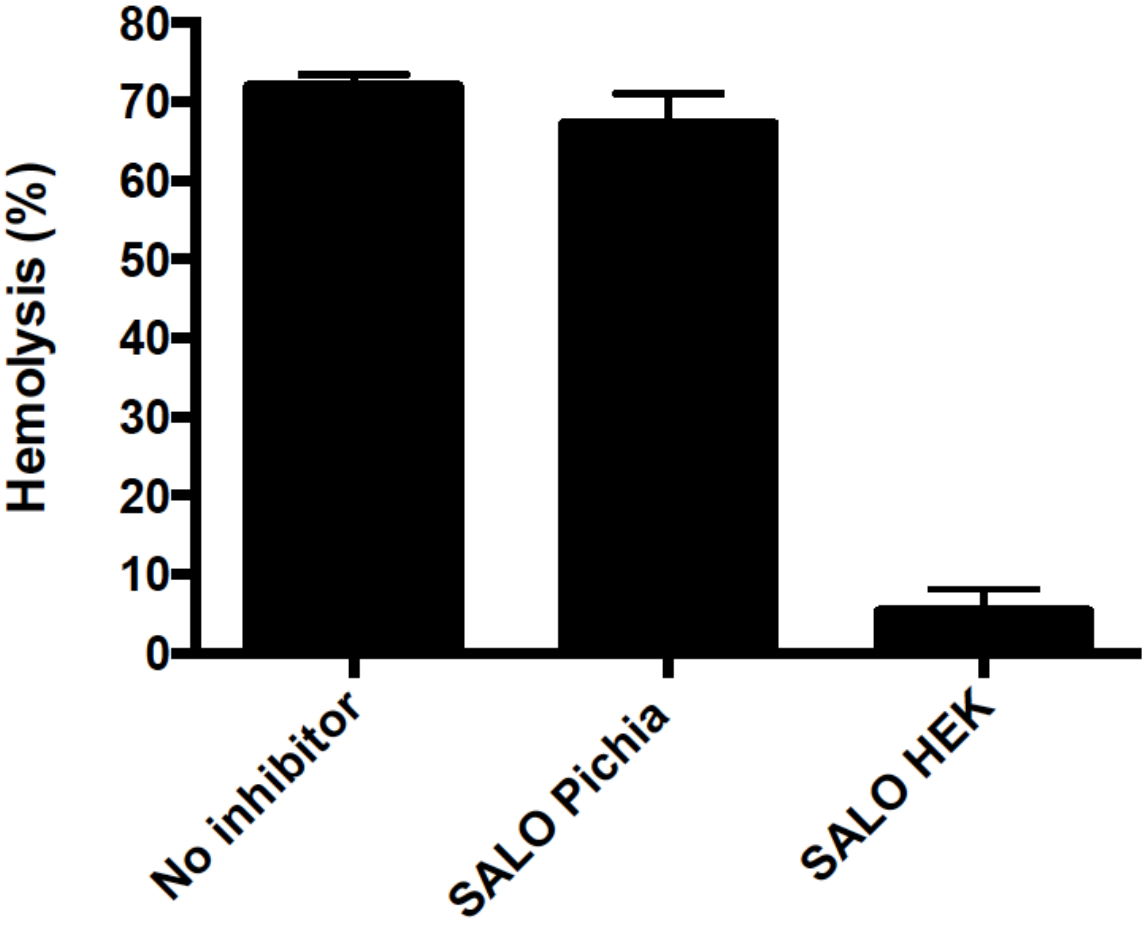

S1 Fig. Recombinant SALO produced in *Pichia pastoris* (rSALO[P]) does not inhibit the classical pathway of complement. rSALO (0.1μM) produced in Pichia (SALO Pichia) or rSALO (0.1μM) produced in HEK cells (SALO HEK) were tested on the classical pathway of complement using a hemolytic assay. Erythrocyte lysis was measured at 414nm. The data represents the mean plus the standard deviation of three independent experiments.
