# Supplementary material for: Structure of SALO, a leishmaniasis vaccine candidate from the sand fly Lutzomyia longipalpis
Source: PLoS Negl Trop Dis. 2017 Mar 9;11(3):e0005374. doi: 10.1371/journal.pntd.0005374 (PMC5344329; doi:10.1371/journal.pntd.0005374)
Supplement: S2 Fig — C57bl/6 mice were immunized in the ear with 2 μg of recombinant SALO produced in P. pastoris (p) or SALO produced in HEK cells (m). Two weeks (A) or 4 weeks (B) after the last immunization 2 μg of recombinant protein was injected and induration and redness in the ear was measure at 48 hours. Naïve mice (control) were injected with PBS. The data represents the mean ± standard deviation of a representative experiment with 5 mice per group of two independent experiments (ANOVA and Tukey test). (PDF) [file pntd.0005374.s003.pdf]

A

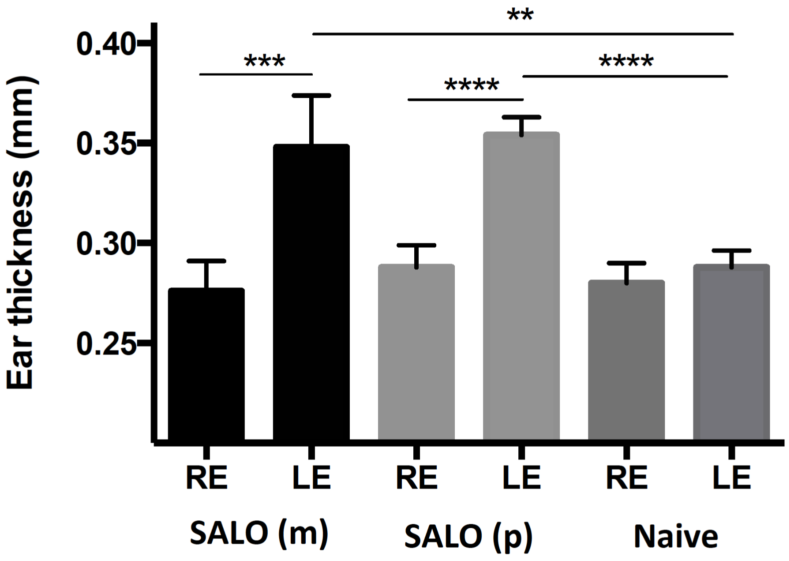


B

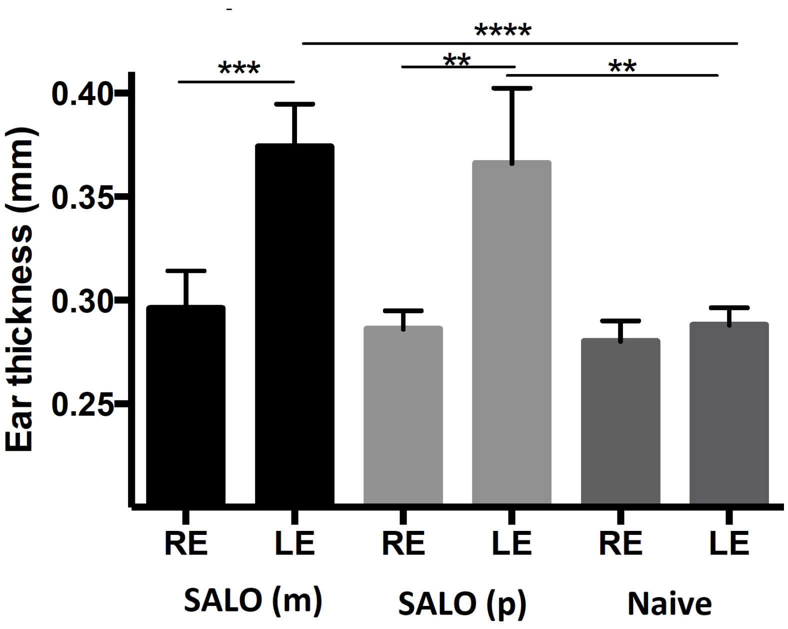


**S2 Fig. Skin immune response in rSALO immunized mice**. C57bl/6 mice were immunized in the ear with 2 μg of recombinant SALO produced in *P. pastoris* (p) or SALO produced in HEK cells (m). Two weeks (A) or 4 weeks (B) after the last immunization 2 μg of recombinant protein was injected and induration and redness in the ear was measure at 48 hours. Naïve mice (control) were injected with PBS. The data represents the mean $\underline{+}$ standard deviation of a representative experiment with 5 mice per group of two independent experiments (ANOVA and Tukey test).
